# A Passive Ladder-Shaped FBG Sensor Network with Fault Detection Using Time- and Wavelength-Division Multiplexing

**DOI:** 10.3390/s25144261

**Published:** 2025-07-09

**Authors:** Keiji Kuroda

**Affiliations:** Department of Physics, School of Science, Kitasato University, Sagamihara 252-0373, Kanagawa, Japan; kkuroda@kitasato-u.ac.jp

**Keywords:** fiber Bragg grating, ladder topology, DFB laser array, time-division multiplexing, wavelength-division multiplexing

## Abstract

This article reports on the interrogation of fiber Bragg grating (FBG)-based sensors that have been multiplexed in a ladder topology. In each line of this topology, FBGs with different wavelengths are connected. In addition, delay fibers have been inserted between each line to enable reflections from different lines to be distinguished. Seven FBGs are interrogated simultaneously by applying time- and wavelength-division multiplexing techniques. To improve the signal-to-noise ratio of the weak reflected signals, the heterodyne detection technique is applied. Through the simulation of three different failure cases, we evaluate the fault detection capability of our method.

## 1. Introduction

Quasi-distributed large-scale fiber sensing has advanced in recent years for use in various applications, including measurement of physical quantities, structural health monitoring, and civil engineering. When used as a sensor head in fiber sensing networks, the fiber Bragg grating (FBG) has attractive properties that include its small size, low losses, light weight, and a flexible design capability. To evaluate the performance of these FBG-based sensor networks, the resolution, sensitivity, multiplexing capability, measurable range, and robustness of each network must be considered. To provide a large-scale multiplexing capability, the number of sensor heads that can be interrogated is increased using techniques including time-division multiplexing (TDM) [1,2,3], wavelength-division multiplexing (WDM) [4,5,6], and a combination of both methods [6,7,8]. The two multiplexing techniques are usually based on the use of a single fiber line arrangement because of its simplicity and power usage efficiency. In TDM schemes, pulsed light sources or optical gates are used to enable reflections from nearly identical FBGs to be distinguished temporally. However, even when FBGs with low reflectivity are incorporated, the up/downstream light fields decrease for the more distant sensor heads, which inevitably results in low signal-to-noise ratios (SNRs). In WDM schemes, broadband light sources are used to cover the wavelength range over which the FBGs can be tuned. Therefore, the number of multiplexing units is limited by the bandwidths of the light sources used.

In operational situations, particularly in harsh environments, the sensor heads can be damaged easily by environmental factors. To increase the reliability of the sensor network, fault detection and the network’s self-healing capability are very important. For this purpose, various network topologies have been proposed in addition to the single line [9]. Several groups have reported multi-ring architectures, in which FBGs that were connected via sub-rings were accessed from two directions using optical switches [10,11,12]. Resilient double-ring networks were realized by incorporating working and protection-type transmission fibers in parallel [13]. Semicircular networks have been proposed and evaluated to enable the construction of networks with a self-healing capability [14,15,16]. A ladder topology has also been proposed to realize robust networks with fault detection. In [17], FBGs were connected into the transmission lines in a ladder topology. In [18,19], FBGs were incorporated within the independent parallel fiber lines of a ladder-shaped network. Both authors claimed that if trouble occurred at a specific point in the network, the trouble spots could be identified and the sensor heads could then be accessed from the opposite direction. In general, WDM schemes have been used in combination with optical spectrum analyzers in the research described above. In [19], the authors reported application of a TDM/WDM technique to a sensor network with fault detection using broadband short pulses. A four-stage ladder network was realized using 75:25, 66:34, and 50:50 couplers. This use of couplers with different ratios enabled sufficient optical intensities to be sent to distant lines. Therefore, multiplexing, fault detection, and multi-access capabilities will be required to realize large-scale, robust sensor networks.

In a previous paper [20], we proposed a heterodyne detection-based technique that allowed the sensitive detection of weak reflected signals. In this heterodyne detection (HD) scheme, reflections can be observed as amplitudes by mixing them with a strong reference field. Therefore, reductions in the signal intensity caused by losses in the networks are reduced when compared with the corresponding losses in the conventional intensity detection (ID) scheme. This advantage is expected to be valid in robust systems when using a ladder topology containing numerous optical couplers. This paper reports on the interrogation of FBGs that have been temporally and spectrally multiplexed in a ladder topology. Seven FBGs with three different wavelengths are connected in three parallel lines in the ladder structure. Reflections from FBGs in the same line are spectrally distinguished. Reflections from FBGs with the same operating wavelength in different lines can be distinguished temporally. The HD technique is used to increase the SNRs for weak reflections from the FBGs. By applying the TDM technique, two reflections from the FBGs propagating in opposite directions are detected in the time domain simultaneously. This facilitates the fault detection and self-healing operation to be performed without optical switches.

## 2. Heterodyne Detection

In HD, a directly modulated distributed feedback laser diode (DFB LD) generates pulse trains [20,21]. These pulse trains are divided into signal pulses, which are sent to the FBG, and reference pulses. These pulses have a frequency drift that is caused by thermal diffusion in the modulated LD. When the optical path length between the two pulses (which have amplitudes of *E*_sig_ and *E*_ref_) differs, the resulting interference between the two pulses produces heterodyne beats with an amplitude of 2|*E*_sig_*E*_ref_|. Even when the reflections from the FBG are very weak, the beat amplitude will be enhanced by the strong reference amplitude. The beat signal that can be obtained using a balanced detector (BD) is written as follows:

I = 2α*R*^1/2^(λ)*E*_sig_*E*_ref_ cos(Δ*ω*t − Δ*ϕ*), (1)

where *α* is a factor that incorporates *ε*_0_*c*/2, the detection efficiency, and the gain of the BD, and the total loss within a fiber line. Here, *ε*_0_ and *c* are the permittivity and the speed of light, respectively. Δ*ω* and Δ*ϕ* represent the angular frequency of the beat signal and the phase difference between the two pulse fields, respectively, and *R*(*λ*) is the reflectivity of the FBG. By calculating an area of *I*^2^ over the pulse width, we obtain

*I*^2^_sum_ = 2α^2^*R*(λ)*I*_sig_*I*_ref_ Σ[1 + cos(2Δ*ω*t − 2Δ*ϕ*)]. (2)

The FBG’s reflectivity *R*(λ) can be defined from this calculation directly. Thus, by measuring the wavelength dependence of *I*^2^_sum_, we can obtain a reflection spectrum of the FBG. Application of the HD technique and use of a BD enhance the SNRs of the FBG’s reflection spectra by more than three orders of magnitude when compared with the ID approach [21]. This enhancement of the SNR facilitates the sensitive detection of reduced reflection signals caused by the use of optical couplers in complex sensor networks.

## 3. Experimental

### 3.1. Interrogator

**Figure 1:**
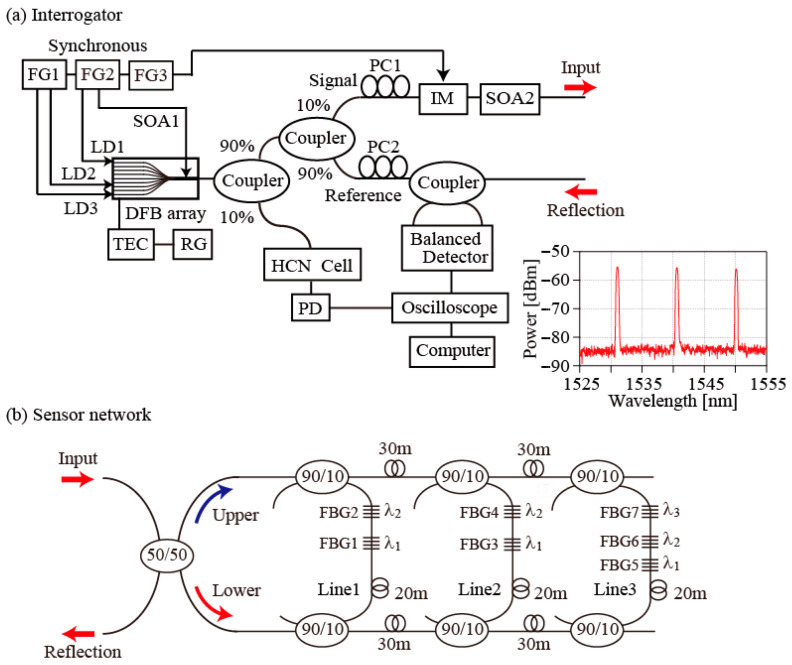
(**a**) Schematic of the experimental setup. LD: laser diode; SOA: semiconductor optical amplifier; DFB array: distributed feedback laser array; FG: function generator; FBG: fiber Bragg grating; PC: polarization controller; PD: photodetector. Inset: Spectra of FGB5, 6, and 7. (**b**) Schematic showing the details of the ladder topology sensor network.

Figure 1a shows the experimental setup used to perform the current work. The light source is a DFB laser array (NLK1CMBLG, NTT Electronics, Yokohama, Japan) comprising 12-channel LDs, a coupler, and a semiconductor optical amplifier (SOA). The operating range of this array spans a wavelength range from 1530 nm to 1560 nm. The optical fields generated by the LDs are all amplified by the SOA1. In the experiments, LD1, which operates at 1531.0 nm, LD2, which operates at 1540.5 nm, LD3, which operates at 1550.1 nm, and the SOA1 are all modulated directly by two synchronized function generators (AFG3252, Tektronix, Tokyo, Japan) (FG1 and FG2). The DFB array’s output is then divided using a 90:10 coupler. To enable the observation of spectral markers, a 10% proportion of the array’s output is input into a H^13^C^14^N fiber-coupled cell. The cell’s transmission is then detected directly using an amplified photodetector (PDA10CS-CE, Thorlabs, Newton, NJ, USA) (PD). The remainder of the output is divided again using a 90:10 coupler to produce signal (10%) and reference (90%) pulses. The signal pulse is shortened using an intensity modulator (FTM7929FB, Fujitsu, Kawasaki, Japan) (IM) controlled by a function generator (FG3) that is synchronized to FG1 and FG2. A polarization controller (PC1) then polarizes the input pulse to be oriented parallel to the modulator’s axis. The shortened signal pulse is introduced into a fiber connected to a ladder topology after being amplified by a second SOA (SOA2), which compensates for the losses that occurred at the IM. The reflections from the FBGs are combined with the reference pulse via a 50:50 coupler. To enable the more effective detection of the resulting beat signals, controller PC2 is used to adjust the reference pulse polarization. The two outputs from the second coupler are detected using a BD (PDB480C-AC, Thorlabs, Newton, NJ, USA). Finally, the interference signals from the BD and the absorption spectra from the PD are monitored using an oscilloscope (PXI-5152, National Instruments, Austin, TX, USA) and the results are stored on a computer. To measure the reflection spectra of the FBGs, the DFB temperature is scanned using a temperature controller (TED200C, Thorlabs, Newton, NJ, USA); this controller is regulated via a ramp generator (Wave station, Teledyne Lecroy, Chestnut Ridge, NY, USA) that outputs a ramp voltage with a period of 30 s.

### 3.2. Sensor Network

**Figure 2:**
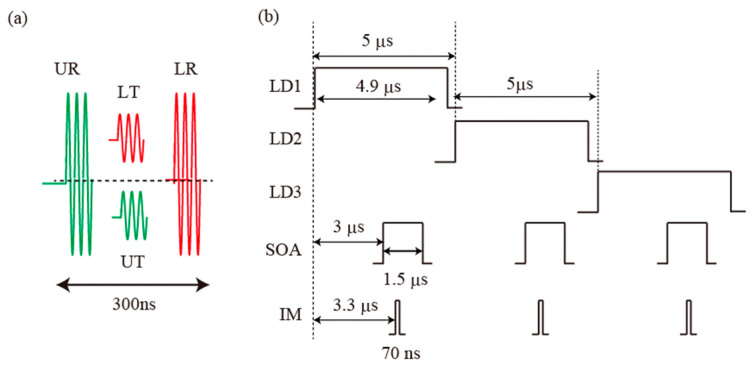
(**a**) Schematic representation of the detected beat signals. UR: reflection from the upper line; LR: reflection from the lower line; UT: transmission from the upper line; LT: transmission from the lower line. (**b**) Time chart for the voltages applied to the LDs, the SOA, and the IM.

The sensor part of the network is a three-line ladder topology comprising one 50:50 coupler and six 90:10 couplers. Two outputs from the 50:50 coupler are introduced into each line via the upper and lower main lines. Seven FBGs are incorporated into each line. These FBGs have a bandwidth of 0.2 ± 0.1 nm, reflectivity of 92%, and sidelobe suppression of more than 20 dB. The peak wavelengths of the groups (FBG1, FBG3, FBG5), (FBG2, FBG4, FBG6), and (FBG7) are *λ*_1_ = 1531.0 ± 0.1 nm, *λ*_2_ = 1540.5 ± 0.1 nm, and *λ*_3_ = 1550.1 ± 0.1 nm, respectively, and they correspond to the wavelengths of LD1, LD2, and LD3. FBG1−4 and FBG5−7 were fabricated by Shinko Electric Wire, Sanuki, Japan and O/E Land Inc, Montreal, Quebec, Canada, respectively. Typical spectra of FBG5, BG6, FBG7 observed using a broadband source and an optical spectrum analyzer (OSA) are shown in the inset of Figure 1a. FBG1 and FBG2, FBG3 and FBG4, and FBG5 to FBG7 are connected to Line1, Line2, and Line3, respectively. Therefore, reflections from FBGs within the same line can be spectrally distinguished using WDM. At a reflection port of the 50:50 coupler, two reflections and two transmissions from the upper and lower lines are obtained in the time domain. In addition to the lengths of the FBG areas (of approximately 5 m), 20 m long fibers are connected between the FBG areas and the lower couplers. The roundtrip times of the reflections from the upper lines, the transmissions from both lines, and the reflections from the lower lines are less than 50 ns, about 125 ns and 200 ns, respectively. Figure 2a shows a schematic representation of the beat signals that are detected in the time domain. The reflections from the upper lines (UR), the transmissions from both lines (LT, UT), and the reflections from the lower lines (LR) are detected in series. The 20 m long fibers are used to temporally separate the upper reflections from the lower reflections. To avoid overlapping of these signals, the signal pulse widths were set to be 70 ns. This means that bi-directional access to the FBGs is possible. Moreover, 30 m long fibers (the roundtrip time of 300 ns) are connected between the couplers. This configuration allows reflections from FBGs with the same wavelength in different lines to be temporally distinguished. In this network, 10% of the incident pulses are introduced into the lines, and only 10% of the reflected pulses are sent back to the central office via the coupler. This means that only 0.23% of the input fields are returned to the detection port. To compensate for the low signal level, the HD technique is applied, rather than use of an optical spectrum analyzer.

### 3.3. Voltages Applied to the Devices

**Figure 3:**
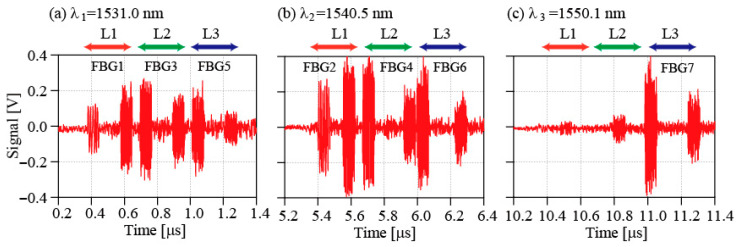
(**a**) Beat signals of FBG1, FBG 3, and FBG 5. (**b**) Beat signals of FBG2, FBG4, and FBG7. (**c**) Beat signals of FBG7.

Figure 3b shows time charts for the voltages that were applied to the LDs, the SOA, and the IM. A voltage pulse with a width of 4.9 µs was applied to LD1 from FG1. A voltage pulse with a width of 4.9 µs and a delay of 5 µs and a voltage pulse with a width of 4.9 µs and a delay of 10 µs were applied to LD2 and LD3 by FG1 and FG2, respectively. Three more pulses with a width of 1.5 µs, an interval of 5 µs, and a delay of 3 µs were applied to the SOA by FG2. Three pulses with a width of 70 ns, an interval of 5 µs, and a delay of 3.3 µs were applied to the IM by FG3. As a result, three-wavelength reference pulses with a width of 4.9 µs and three-wavelength signal pulses with a width of 70 ns were generated. The interval between these pulse groups was set at 1 ms.

## 4. Results

### 4.1. No Fault Case

**Figure 4:**
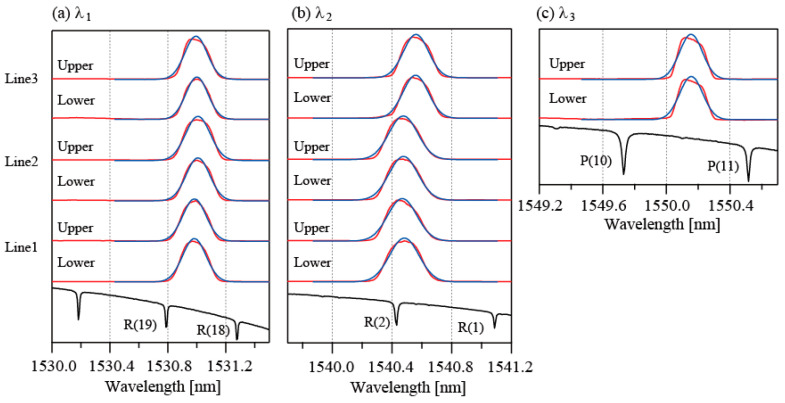
(**a**) Spectra of FBG7, FBG3, and FBG5 as observed in the upper and lower lines, along with the absorption spectrum of HCN. (**b**) Spectra of FBG6, FBG2, and FBG4, along with the absorption spectrum of HCN. (**c**) Spectrum of FBG1 along with the absorption spectrum of HCN. The blue lines represent the fitting results.

Figure 3a–c show the beat signals obtained for the three wavelengths when the wavelengths of the LDs were tuned to be close to the central wavelengths of the FBGs. At each wavelength, three signal groups (L1, L2, and L3) are observed sequentially. In each group, two beat signals are detected. These signals correspond to the upper reflections and the lower reflections, respectively. Because the FBG reflectivities are high, transmissions between the reflection beats almost disappear, except for those in groups L1 and L2 for *λ*_3_. Figure 4a–c show the spectra as calculated from the normalized *I*^2^_sum_ as a function of the wavelength. The spectra are shifted vertically in the figure for clarity. Two spectra obtained from the upper and lower lines are shown for each FBG in the figures. The lower part in each figure shows the absorption spectrum of H^13^C^14^N. Six absorption lines, comprising P (19) at 1530.78615 nm and P (18) at 1531.27537 nm, R (2) at 1540.43120 nm and R (1) at 1541.08703 nm, and P (10) at 1549.73051 nm and P (11) at 1550.51546 nm, were used as wavelength markers to determine the peak wavelengths [22]. The abscissas in these figures were calculated using the marker wavelengths. The SNRs are confirmed to be greater than 10 dB for all spectra. We believe that this SNR is sufficient to determine the peak wavelength from the spectra. The blue lines represent the fitting results that were obtained using a Gaussian function. When the reflectivity increases, the reflection spectrum will then generally deviate from the Gaussian function. In this study, FBGs with a reflectivity of 92% were used. This high reflectivity accounts for the disagreement between the experimental data and the fitting lines. Therefore, we only used the fitted curves to determine the peak wavelengths of the FBGs. The peak wavelengths that were calculated from the curve fittings agree well with the specifications to within ±0.1 nm. We have confirmed that our method can be used to measure the peak wavelength of FBGs over a temperature range of 50 °C and determined coefficients to be about 0.011 nm/°C [21,22]. This result indicates that by applying the HD technique to increase the signal intensities of the weak reflections, bidirectional access to the FBGs that are incorporated in the ladder-shaped network can be performed without using mechanical devices such as an optical switch.

### 4.2. Fault Detection

**Figure 5:**
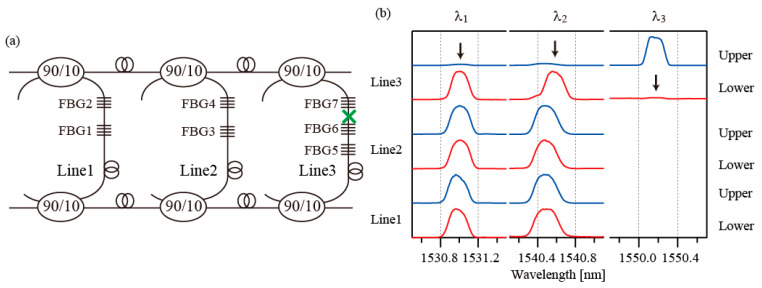
(**a**) Fault case 1. The green cross indicates the fault position. (**b**) Spectra of the FBGs. Black arrows indicate the positions of spectra that have disappeared.

In this section, to assess the network’s robustness, the interrogation results in three fault cases are presented. In the first fault case, the fault was assumed to occur at a point between FBG6 and FBG7, as indicated by the green cross in Figure 5a. The observed spectra are shown in Figure 5b. The blue lines and red lines represent the spectra acquired from the upper and lower lines, respectively. Arrows indicate the positions where the spectra disappeared. The spectra of FBG7 in the upper line and those of FBG6 and FBG5 in the lower line have disappeared. From these observations, the fault position in the network can be identified. The spectra of FBG7 and (FBG6, FBG5) can be observed from the upper and lower lines, respectively. This indicates that this network configuration is robust to the occurrence of a single fault inside each line.

**Figure 6:**
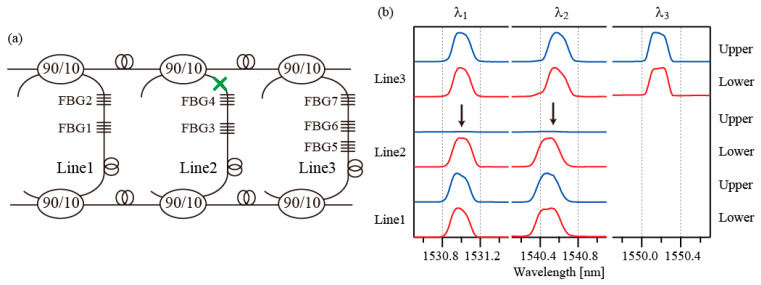
(**a**) Fault case 2. The green cross indicates the fault position. (**b**) Spectra of the FBGs. Black arrows indicate the positions of spectra that have disappeared.

**Figure 7:**
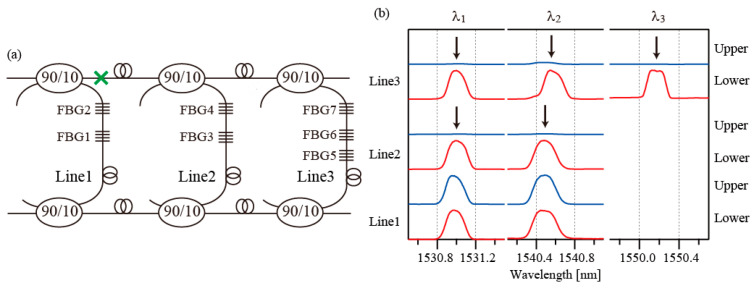
(**a**) Fault case 3. The green cross indicates the fault position. (**b**) Spectra of the FBGs. Black arrows indicate the positions of spectra that have disappeared.

In the second fault case, the fault was assumed to occur at a point between FBG4 and the upper line coupler, as indicated by a green cross in Figure 6a. The observed spectra are shown in Figure 6b. The spectra of FBG3 and FBG4 are missing from the upper line. However, the two FBGs can still be accessed from the lower line. In the third fault case, the fault was assumed to occur after the first coupler in the upper main line, as shown by the green cross in Figure 7a. The observed spectra in this case are shown in Figure 7b. The spectra of Line2 and Line3 are all missing from the upper line. In this case, the upper main lines used to guide the optical signals are broken. However, all FGBs can still be accessed through the lower main line.

## 5. Discussion

In a review paper [7], the requirements for construction of robust optical fiber sensor networks were discussed. The three criteria that must be fulfilled simultaneously were provided as follows: 1: the network must be able to withstand at least one fiber failure at any point; 2: the network must operate with nominally equal transmission losses for all sensing channels when passing from transmitter mode to receiver mode, both during normal operation and after recovery from a failure; 3: it must be possible to signal a failure and take the required actions without using external resources such as dedicated fiber or radio links. The results presented in Figure 5, Figure 6 and Figure 7 show that criteria 1 and 3 are fulfilled. When a single fault occurs at any point in the main lines or the sensing lines, all the FBGs still survive. In addition, the fault positions can be identified from the 14 spectra.

**Figure 8:**
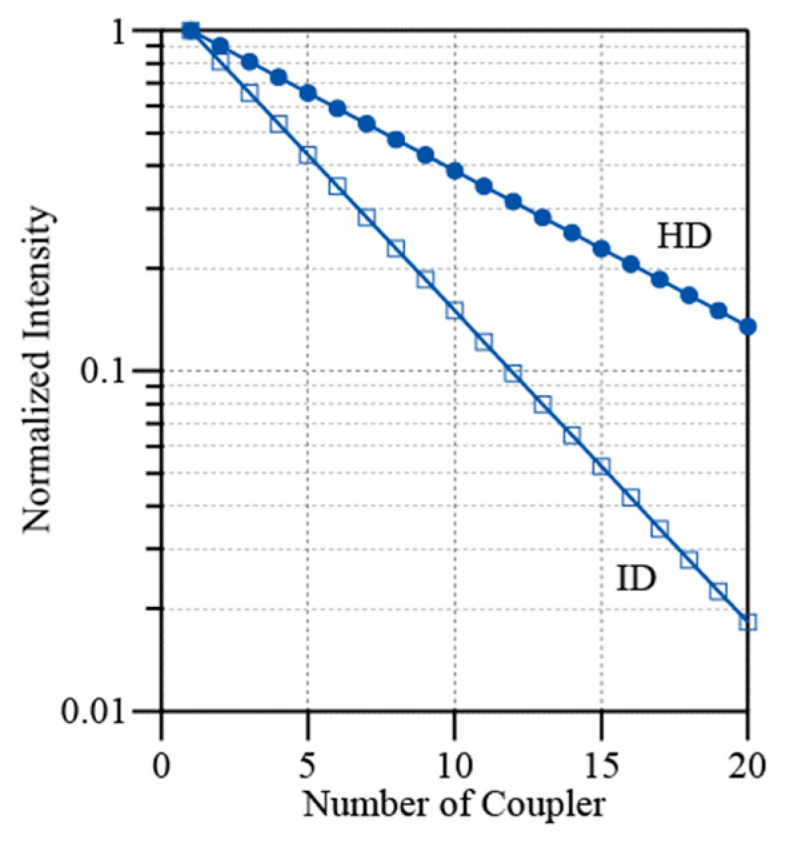
Dependences of the normalized intensity on the number of couplers for both ID (squares) and HD (circles).

In the ladder topology, the sensor heads are incorporated into lateral lines using fiber couplers. If it is assumed that all the FBGs have the same reflectivity *R*, and that the coupler excess loss is negligible, then the reflection intensity of the FBGs from the *n*th line is expressed as *I*_ID_ = |*E*_sig_|^2^*Rk*^2^(1 − *k*)^2(*n*−1)^. Here, *k* is the coupling ratio of the coupler and it is set at 0.1. Because the reflections from the FBGs are detected as 2|*E*_sig_*E*_ref_| in HD, the equation is rewritten as *I*_HD_ = 2|*E*_sig_*E*_ref_|*R*^1/2^*k*(1 − *k*)^(*n*−1)^. With respect to the intensities normalized by the intensity of the FBGs in the first line, these expressions are modified to read *I*_ID_ = (1 − *k*)^2(*n*−1)^ and *I*_HD_ = (1 − *k*)^(*n*−1)^, respectively [23]. Figure 8 illustrates the coupler number dependences of the normalized intensities for both ID (open squares) and HD (circles). We see that the normalized intensity from the FBGs in the 20th branch is reduced by approximately 0.015 when compared with the intensity of the first FBG for ID. In contrast, the corresponding reduction for the HD case is approximately 0.12. Although the transmission losses of the FBGs are not completely equal, the signal intensities are of the same order up to the 20th coupler. When criterion 2 is considered, our method is advantageous when the number of lines in the ladder topology is increased.

Because the coupler loss and the loss between FBG fibers are neglected in the above calculation, we consider the expansion of the system up to 10 lines as a realistic situation. In this setup, the delay length between the couplers is 30 m. Even when we expand the system up to 10 lines, a total roundtrip length is less than 1 km. Giving that the propagation loss of silica fiber is 0.2 dB/km, the propagation loss is still negligible. A width required to detect beat signals from a single FBG is 300 ns. Thus, a pulse width longer than 3 μs is required for the reference fields. This is easily achievable by adjusting conditions of the applied voltages indicated in Figure 2b. The DFB laser can cover the wavelength range from 1530 nm to 1560 nm with 11 LDs. Therefore, a high-density sensor line with a multi-channel pulse generator and 11-wavelength FBGs inscribed in a single fiber can be constructed based on the system proposed in this work. In the WDM sensing, reflections from different-wavelength FBGs are observed in mixing with different reference fields as can be seen in Figure 3. This indicates that intervals between FBGs in the same line do not affect the beat signals. Thus, denser spacing of the order of a few cm can be realized.

Table 1 shows a comparison of our system with those in previous studies. In refs. [15,16], the authors applied the WDM technique with an OSA. Thus, measurement accuracies were limited by the resolution of the OSAs. The number of sensor heads was determined by bandwidths of light sources. In ref. [17], the authors applied the WDM/TDM hybrid technique using a broadband pulse source, a wavelength-division multiplexed demodulator, and an optical time-division visualizer. In their setup, peak wavelengths of FBGs are determined in the time-domain using short pulses with a pulse width shorter than a nanosecond. Although they did not discuss the resolution of the system, the high-resolution sensing may be difficult. The advantages of our method are that (i) high-resolution measurement is possible using the molecular markers, (ii) the setup is relatively simple without OSA and optical switch, and (iii) the system can be expanded to larger scale. To date, related studies including this work have treated a small number of FBGs. As shown in Figure 8, I expect that our method based on HD is more suitable to the interrogation of larger-scale ladder networks.

## 6. Conclusions

This paper reported on the interrogation of FBGs that have been temporally and spectrally multiplexed in a ladder topology. Seven FBGs with three different wavelengths were connected via three parallel lines in the ladder. Reflections from FBGs in the same line were distinguished spectrally via WDM. Reflections from FBGs with the same operating wavelength in different lines were distinguished temporally. The HD technique was used to increase the SNRs for weak reflections from the FBGs. By applying the TDM technique, two reflections from the FBGs traveling in opposite directions were detected simultaneously in the time domain. This allowed the fault detection and self-healing operation to be performed without optical switches.

## Figures and Tables

**Table 1:** Comparison of this work and previous reports.

	Ref. [15]	Ref. [16]	Ref. [17]	This Work
Source	^1^ EDF	^1^ EDF	Broadband Pulse	DFB LD
Multiplexing	WDM	WDM	WDM/TDM	WDM/TDM
First-Line SNR	20 dB	30 dB	30 dB	20 dB
FBG	6	8	8	7
Detection	OSA	OSA	^2^ WDMD ^3^ OTDV	BD
Resolution	60 pm	10 pm		3 pm [24]
Bi-directional access	Optical switch	Optical switch	Optical switch	Coupler

^1^ EDF: erbium-doped fiber; ^2^ WDMD: wavelength-division multiplexed demodulator; ^3^ OTDV: optical time-division visualizer.

## Data Availability

Data can be provided upon request.

## Abbreviations

The following abbreviations are used in this manuscript:

DFB-LD	Distributed feedback laser
FBG	Fiber Bragg grating
TDM	Time-division multiplexing
WDM	Wavelength-division multiplexing
OSA	Optical spectrum analyzer
HD	Heterodyne detection
ID	Intensity detection
BD	Balanced detector
SOA	Semiconductor optical amplifier
PD	Photodetector
IM	Intensity modulator
FG	Function generator
PC	Polarization controller
SNR	Signal-to-noise ratio
EDF	Erbium-doped fiber
WDMD	Wavelength-division multiplexed demodulator
OTDV	Optical time-division visualizer
